# Editorial: Outside the Comfort Zone: What Can Psychology Learn From Tourism (and Vice Versa)

**DOI:** 10.3389/fpsyg.2021.650741

**Published:** 2021-03-25

**Authors:** Paola Passafaro, Claudia Chiarolanza, Clara Amato, Barbara Barbieri, Elena Bocci, Mauro Sarrica

**Affiliations:** ^1^Department of Developmental and Social Psychology – Sapienza University of Rome, Rome, Italy; ^2^Department of Dynamic and Clinical Psychology, and Health Studies – Sapienza University of Rome, Rome, Italy; ^3^Landstinget Blekinge Karlskrona, Karlskrona, Sweden; ^4^Department of Political and Social Sciences – University of Cagliari, Cagliari, Italy; ^5^Department of Communication and Social Research – Sapienza University of Rome, Rome, Italy

**Keywords:** tourism psychology, tourism risk perception, attachment to tourism places, tourism experience, museum experience

Today, tourism represents a pivotal and not interchangeable social practice that can move substantial individual, social, cultural, environmental and economic forces (and resources) worldwide. In 2019, the World Tourism Organization indicated tourism as “one of the major players in international commerce with a business volume that equals and even surpasses that of oil exports, food products or automobiles” (UNWTO, 2019). Tourism provided a job to one-tenth of the global population and has become the main opportunity for employment in many developing countries. This explains why the COVID-19 pandemic, by halting the tourism industry, has contributed to the emergence of an unexpected economic crisis in many countries (Grech et al., 2020; Ugur and Akbiyik, 2020). Such crises have also dramatically revealed tourism's tight association with many cogent issues of social, cultural and environmental relevance including cultural heritage conservation, social equity and inclusiveness, human and environmental exploitation, diversity and mutual understanding, peace, safety, and security.

Building on the work of tourism scholars and practitioners over many years of research, it is, thus, even more evident now that tourism overlaps with a number of topics at the center of psychological research, including quality of life, well-being, personal health and resilience, close-relationships and sociality, community and place relationships, environmental threat and biodiversity conservation, mass communication, and marketing and e-commerce (and many more). For this reason, it involves numerous research areas within psychology, from the social to work and organizational psychology, from environmental to economic psychology, from health to family psychology and positive psychology, and so forth. Nevertheless, despite its central role in modern Western societies, tourism has always encountered serious difficulties in being accepted as a relevant area of scientific interest in the field of psychology (e.g., Pearce, 1987; Berno and Ward, 2005). The reasons for this might lie, in part, in the apparent superficiality of the topic, which is connected with leisure and amusement, and it is often seen as part of the superfluous and marginal moments of individual and social life (Pearce, 1987). The aura of apparent playfulness associated with this theme may have obscured, in the eyes of some researchers, the intrinsic seriousness of the many tourism-related issues, as well as the relevance of investigating them to reach a full understanding of human behavior.

However, other serious theoretical and methodological nature reasons need to be addressed. For example, today, tourism psychology is a fruitful field for experiments in new approaches and perspectives, not all of which are necessarily in line with the mainstream, broader psychological field. This could be due to the applied focus adopted by researchers in tourism that required certain methodological flexibility. The inherent inter and cross-disciplinary vocation of the topic might have induced colleagues in tourism psychology to break away from disciplines of origin, to trace new connections with theories and methods closer to those research areas, which seem to have better understood the importance of studying this particular human behavior (e.g., the fields of economy and geography, and the emerging, managerial engineering). Perhaps, because they were already involved in the demanding process of opening new channels of trans-disciplinary communication, tourism psychologists have demonstrated a tendency to focus more on how psychology can help to address tourism issues, than on how tourism studies can increase our knowledge of psychology. Indeed, it is difficult to find research that attempts to delineate connections in both directions. This represents a serious limitation of the area that needs to be addressed.

The goal of this Research Topic is to try to establish this two-way communicative connection. In particular, we wanted to offer tourism scholars (psychologists and non-psychologists) the opportunity to set studies within the broader psychological field, and to stimulate psychology as a discipline to expand its view on human psychology and behavior by covering a too often neglected area of research.

The articles in this topic issue make the first step in this direction. First, they provide psychologists with a taste of the wide repertoire of specific themes under investigation in tourism studies. They show that a broad range of phenomena exists, which need to be explored, classified and interpreted by psychologists to add important pieces to the puzzle of human experience, drawn by psychology as a whole. Second, they show that virtually any theoretical and methodological perspective can be applied to the investigation of this topic area, from qualitative to quantitative approaches, from case studies to field and experimental studies. Third, the contributions show the usefulness of tourism research as a terrain for inter/cross- disciplinary theoretical reflection and experimentation. The authors in this issue have left their disciplinary comfort zone to apply psychological theories and/or methods in new domains or to respond to unconventional queries for the origin field. This is surely useful for a better understanding of the tourism issues investigated (see Pearce and Packer, 2013), but it is also valuable for the evolution of psychology as a science.

As already stated, tourism issues involve or are linked to most of the main Research Topic areas in psychology, so studies on tourism have the potential to contribute new insights and perspectives to these areas.

For example, increasingly numerous literature has shown that tourism can represent an experiential and behavioral context in itself, different from other contexts, a sort of “other” dimension in which the psychological laws so far identified could present some exceptions, suspensions, or alternative ways of functioning. This was confirmed by the results of the study by Bø and Wolff on the relationship between episodic future thinking (EFT) and risk perception. The authors acknowledge that previous research on the topic in other situational contexts had shown how EFT (a construct related to the ability to think about specific events that can be personally experienced in the future) can affect the individual perception of risks. As the authors point out, this topic is of particular relevance because risk perception represents one of the most important factors, affecting individual decisions to perform risk-related behaviors, and it is necessary to enhance our theoretical understanding of its antecedents. It can also have important practical consequences in various fields, including tourism, where perceived risk has been shown to influence people's decision to travel. Indeed, it is likely that the relevance of specific types of risk perception (e.g., health risks) will play a crucial role in the resumption of tourism after the pandemic. The study by Bø and Wolff adds to the conspicuous literature on the antecedents of risk perception by introducing a case in which EFT seems not to produce the expected effects. The authors propose various theoretical and methodological explanations for their contrasting results, but they also acknowledge that a similar singularity had already emerged in a previous study by Floyd et al. (2004), who found the perceived risk of terrorism to be unrelated to travel intentions. Other studies also seem to support the idea that tourism represents a research area in which psychological theories are severely tested (e.g., Juvan and Dolnicar, 2014, 2017), and this would mean that tourism and leisure studies have the potential to contribute to the evolution of such theories, and the progress of psychological models in general. Another example of this is the article by Shen et al., which explores the intersection of two well-established theoretical traditions: place attachment (a central theme of environmental psychology; Altman and Low, 1992 for recent discussions see Raymond et al., 2010; Scannell and Gifford, 2010; Lewicka, 2011; Manzo and Devine-Wright, 2013; Di Masso et al., 2019; Devine-Wright et al., 2020), and residents' place image, pro-tourism attitudes, and intentions (key topics in consumer and tourism studies). In environmental psychology, this surely represents quite an unedited combination, as the studies on the consequences of place attachment for consumers' choices are still limited. Indeed, in environmental psychology, place attachment has mostly been studied as a key factor involved in individuals' mobility across places (intention to stay or to abandon a place) and in relation to its consequences for the individual and community well-being and/or safety. Another well-established tradition concerns the role place attachment could play on the adaptation of particular social categories (e.g., the elderly, children, migrants, etc.) to new environments or radical changes occurring in known ones. Several studies in the psychological field have also recognized the role of place attachment in place defensive attitudes and behaviors, including pro-environmental behavior. However, the potential of place attachment to explain and predict specific intentions and behaviors related to consumer choices has particularly attracted the attention of researchers in tourism. In this field, a growing number of studies have used this construct to interpret the implications of the emotional and affective bounds developed by tourists *vis à vis* the places visited. The study by Shen and colleagues opens up additional new scenarios, and possibly new queries, in this research area. Unlike previous studies, it focuses on residents, the so called “hosts,” as they are traditionally identified in this field. The study evaluates the role played by place attachment in accepting the transformation of one's own place of residence in a tourist location. The combined focus on place attachment and place image leads the study toward the investigation of unexplored aspects in both research traditions. In particular, for the field of environmental psychology, it raises the question of the role played by the personal and/or shared image (or representation) of a place in the development and evolution of place attachment over time, and looks toward the necessity to deepen the social-cultural correlates and implications of place attachment. This may provide hints on how to shift the theoretical formation of this construct from an exclusively individual-centered perspective to one that takes inter-individual and social factors into greater account. The decision to focus attention on residents/hosts also opens news scenarios in the field of tourism research, as the authors introduce the first measure of the construct from the residents' point of view.

The other two papers in this topic issue by Skavronskaya et al. and by Recupero et al. also provide original contributions to psychological science by focusing on the concept of experience, a core construct in tourism research, the relevance of which has often been overlooked. Indeed, across the various research areas, psychologists have shown a preference for the analysis of psychological processes and factors that are long-lasting and persistent across contexts and situations. The relevance and extent of temporary and/or one-time psychological phenomena have largely been underestimated. Greater attention to these aspects would instead allow us to shed more light on the psychological factors involved in those moments of human life that do not flow along the predetermined tracks of ordinary events. In addition, it would allow us to better put into practice the theoretical reflections of classical authors such as K. Lewin and U. Bronfenbrenner, who highlighted the importance of studying the situational and contextual determinants of human behavior. Indeed, a deep theoretical and methodological reflection on the best ways of framing such contextual and situational factors in psychological terms and on how to incorporate them into psychological models of particular experiences and behaviors is missing. The two research studies by Skavronskaya et al. and Recupero et al. may provide some hints in this direction. Experience, even though personal and intimate, can only be understood by taking into account the context and the situation in which it is generated, and the two papers not only suggest ways to conceptualize and operationalize experience, but they also provide examples on how to integrate it within comprehensive theoretical models. More specifically, the study by Skavronskaia et al. deals with the conceptualization of the experience of novelty and its incorporation into a comprehensive model of the antecedents and consequences of memorable tourism experiences and sense of identity. The authors draw on the theoretical principles of cognitive appraisal theory but propose a reinterpretation of it that integrates phenomenological-hermeneutic components. The study intended to overcome the difficulty of experimental approaches to adequately seize all nuances of personal implications at both the cognitive and emotional level. The use of qualitative methods for investigation, such as semi-structured interviews, also challenges the experimental foundations of cognitive theory in favor of a more holistic analysis of the phenomena investigated.

This type of holistic analysis is also recommended by Recupero et al., who present a psychological study of the factors involved in the experience of museums. The authors respond to the need for a new approach to museum organization and management as a consequence of the social-cultural changes occurring in the conceptualization of a museums' role within contemporary societies. The passage from a collection-centered perspective to an audience-centered one imposes a radical reconsideration of museology as a science and the identification of new theoretical models to analyse and interpret visitor experience. The authors respond to this call by proposing a comprehensive model of investigation and analysis that is also able to respond to the applicative purposes of the field.

The four papers gathered in this Research Topic have the potential to inspire researchers in psychology to dedicate greater attention to tourism studies. This will give us a chance to reduce the paradox of a psychology of tourism made by non-psychologists and to contribute to the consolidation of a theoretically and methodologically grounded travel and tourism psychology.

## Author Contributions

This paper was conceived and written by all the authors conjunctly.

## Conflict of Interest

The authors declare that the research was conducted in the absence of any commercial or financial relationships that could be construed as a potential conflict of interest.
